# Supplementary material for: The Combined Intervention of Aqua Exercise and Burdock Extract Synergistically Improved Arterial Stiffness: A Randomized, Double-Blind, Controlled Trial
Source: Metabolites. 2022 Oct 13;12(10):970. doi: 10.3390/metabo12100970 (PMC9609722; doi:10.3390/metabo12100970)
Supplement: Supplementary file 1 [file metabolites-12-00970-s001.zip › metabolites-1840286-supplementary.pdf]

Table S1. Composition of the burdock root extract.

Water (%)	Crude ash (%)	Crude fat (%)	Crude protein (%)	Crude fiber (%)	Calcium (%)	Phosphorus (%)
98.02±0.02	0.10±0.00	1.12±0.00	0.20±0.00	0.03±	0.004±0.00	0.009±0.00

Pukyong feed & foods nutrition research center

Table S2. Details of the 16-week aqua exercise program for older women with metabolic syndrome.

Weeks	Order	Exercise	Intensity	Frequency
	Warm-up (5min)	Static stretching		
1~5		▸aquarobics step box<br>- step up<br>- side stepping<br>- hopping	30~40 %HRR (RPE 9~10)	
6~10	Main exercise (40min)	▸hydrotone<br>-resistive walking<br>-scissors	40~50 %HRR (RPE 11~12)	3times/week
11~16		▸powerful<br>-fast jogging<br>-kicking	50~60 %HRR (RPE 13~14)	
	Cool-down (5min)	Static stretching		

RPE = Rating of Perceived Exertion

Table S3. Descriptive characteristics of the study participants.

Groups Variables		CON (n=10)	EX (n=10)	BD (n=11)	EXBD (n=11)	P-value
Age (yrs)	M	78.32	77.61	77.14	76.33	0.266
	SD	5.78	3.16	4.23	5.42	
Height (cm)	M	150.62	152.69	153.14	151.85	0.624
	SD	3.28	5.26	4.91	4.23	
Weight (kg)	M	56.15	56.18	59.47	58.24	0.312
	SD	1.85	4.58	7.58	6.68	
WC (cm)	M	90.24	91.87	91.69	90.68	0.723
	SD	4.25	3.24	5.68	8.97	
BMI (kg/m2)	M	24.67	24.75	25.77	25.79	0.238
	SD	0.99	1.25	1.97	2.14	
TG (mg/dl)	M	160.78	169.87	146.89	170.58	0.266
	SD	35.14	37.48	45.58	32.85	
HDL-C (mg/dl)	M	49.57	48.27	48.04	47.82	0.729
	SD	13.98	20.46	16.35	8.18	
Glucose (mg/dl)	M	108.24	114.36	103.89	108.55	0.245
	SD	22.65	20.13	17.28	18.68	
SBP (mmHg)	M	143.68	149.23	158.92	157.78	0.273
	SD	18.74	10.58	26.78	18.68	
DBP (mmHg)	M	72.47	76.25	81.78	81.24	0.074
	SD	6.38	7.65	10.57	4.20	

CON=Control, EX=Exercise, BD=Burdock, EXBD=Exercise+Burdock, WC=Waist circumference, BMI=Body mass index, TG=Triglycerides, HDL-C=High density lipoprotein cholesterol, SBP=Systolic blood pressure, DBP=Diastolic blood pressure. Values are M±SD.

Table S4. Effects of the 16-week aqua exercise on blood pressure and insulin resistance in older women with metabolic syndrome.

Variable	Group	Pre		Post		Cohen's d	Main effect	P-value	Post-hoc
		M	SD	M	SD	Effect size			
SBP (mmHg)	CON (n=10)	143.68	18.74	149.97	11.87	.34	Time	0.039† (F=4.718)	
	EX (n=10)	149.23	10.58	147.58	6.25	.16	Group	0.416	
	BD (n=11)	157.13	24.37	146.18*	17.53	.45	Time*Group	0.122	
	EXBD (n=11)	158.77	18.68	152.36	21.33	.38			
DBP (mmHg)	CON (n=10)	74.38	6.37	75.69	7.15	.21	Time	0.968	
	EX (n=10)	76.91	7.78	76.56	6.56	.05	Group	0.050† (F=2.958)	
	BD (n=11)	81.26	10.47	76.36	9.25	.47	Time*Group	0.643	
	EXBD (n=11)	79.68	6.87	80.25	7.68	.08			
Insulin (mg/dl)	CON (n=10)	19.47	31.05	28.17	27.75	.30	Time	0.603	
	EX (n=10)	32.77	20.73	30.17**	22.76	.12	Group	0.793	
	BD (n=11)	11.24	7.55	21.68	13.17	.97	Time*Group	.003 (F=6.496)	
	EXBD (n=11)	18.75	9.20	13.07	6.77	.70			
Glucose (mg/dl)	CON (n=10)	113.28	22.46	118.77	24.38	.25	Time	0.029† (F=4.765)	
	EX (n=10)	120.24	23.47	95.48***	13.31	1.06	Group	0.179	CON>BD*
	BD (n=11)	97.69	12.58	96.65	11.57	.08	Time*Group	.004†† (F=5.287)	
	EXBD (n=11)	99.57	15.47	86.01*	6.87	.87			
HOMA-IR	CON (n=10)	6.44	11.64	9.87	13.12	.28	Time	0.351	

	EX (n=10)	10.45	7.53	3.39**	1.77	1.29		
							Group	0.673
	BD (n=11)	2.78	2.10	5.18	3.24	.88		
	EXBD (n=11)	5.18	3.77	2.87	1.57	.80	Time*Group	.002 (F=6.972)
QUICKI	CON (n=10)	0.62	0.44	0.21**	0.12	1.27	Time	0.410
	EX (n=10)	0.14	0.82	0.41*	0.27	.44		
							Group	0.689
	BD (n=11)	0.51	0.28	0.29	0.22	.87		
	EXBD (n=11)	0.31	0.25	0.48	0.32	.60	Time*Group	.002 (F=7.246)

CON=Control, EX=Exercise, BD=Burdock, EXBD=Exercise+Burdock, SBP=Systolic blood pressure, DBP=Diastolic blood pressure, HOMA-IR=Homeostatic Model Assessment for Insulin Resistance, QUICKI=Quantitative Insulin Sensitivity Check Index. Values are M±SD. Note: †p<0.05.*p<0.05, **p<0.01, ***p<0.001vs.pre-trial. Effect size range: |.20|≦small<|.50|<medium<|.80|≦large.

Table S5. Changes in blood pressure and insulin resistance after the 16-week aqua exercise in older women with metabolic syndrome.

Variable	Group	M	SD	Source		
				F-value	P-value	Post-hoc
△SBP (mmHg)	CON (n=10)	-7.00	15.14	2.328	0.105	
	EX (n=10)	10.50	6.80			
	BD (n=11)	17.06	25.11			
	EXBD (n=11)	10.33	19.01			
△DBP (mmHg)	CON (n=10)	-2.29	8.98	0.568	0.643	
	EX (n=10)	-3.33	16.03			
	BD (n=11)	6.40	18.09			
	EXBD (n=11)	-0.33	10.29			
△Insulin (mg/dl)	CON (n=10)	-8.70	8.63	6.793	0.002††	CON<EX* CON<EXBD* EX>BD* BD<EXBD*
	EX (n=10)	6.56	7.13			
	BD (n=11)	-10.44	11.05			
	EXBD (n=11)	5.68	6.99			
△Glucose (mg/dl)	CON (n=10)	-9.57	12.37	5.446	0.007††	CON<EX**
	EX (n=10)	26.97	25.91			
	BD (n=11)	-0.02	11.52			
	EXBD (n=11)	14.67	15.59			
△HOMA-IR	CON (n=10)	-3.43	2.79	7.566	0.001††	CON<EX** CON<EXBD* EX>BD*
	EX (n=10)	4.04	4.41			
	BD (n=11)	-2.40	2.68			
	EXBD (n=11)	2.32	2.82			
△QUICKI	CON (n=10)	0.42	0.36	6.427	0.003††	CON<EX* CON>EXBD**
	EX (n=10)	-0.07	0.09			
	BD (n=11)	0.23	0.32			
	EXBD (n=11)	-0.17	0.21			

CON=Control, EX=Exercise, BD=Burdock, EXBD=Exercise+Burdock, BP=Blood Pressure, HOMA-IR=Homeostatic Model Assessment for Insulin Resistance, QUICKI=Quantitative Insulin Sensitivity Check Index. Values are M±SD. Note: †p<0.05, ††p<0.01; difference value than pre-test.

Table S6. Effects of the 16-week aqua exercise on vascular regulation factors in older women with metabolic syndrome.

Variable	Group	Pre		Post		Cohen's d	Main effect	P-value
		M	SD	M	SD	Effect size		
PGI_2 (pg/ml)	CON (n=10)	20.69	5.21	20.52	5.29	.03	Time	0.237
	EX (n=10)	15.98	2.95	19.32*	6.43	.67	Group	0.596
	BD (n=11)	23.53	9.51	23.45	9.42	.01		
	EXBD (n=11)	20.89	8.93	21.85	9.86	.10	Time*Group	0.422
TXA_2 (pg/ml)	CON (n=10)	22.43	6.32	24.73**	6.69	.35	Time	0.505
	EX (n=10)	18.39	5.11	16.88*	4.48	.31	Group	0.377
	BD (n=11)	22.77	7.77	22.83	7.60	.01		
	EXBD (n=11)	22.42	6.66	20.56*	5.88	.30	Time*Group	0.002†† (F=7.207)

CON=Control, EX=Exercise, BD=Burdock, EXBD=Exercise+Burdock, PGI_2=Prostaglandin I_2, TXA_2=Thromboxane A_2. Values are M±SD. Note: ††p<0.01. *p<0.05, **p<0.01 vs pre-trial. Effect size range: ∣.20∣≦small<∣.50∣<medium<∣.80∣≦large.

Table S7. Changes in the vascular regulation factors after the 16-week aqua exercise in older women with metabolic syndrome .

Variable	Group	M	SD	Source		
				F-value	P-value	Post-hoc
△PGI_2 (pg/ml)	CON (n=10)	0.17	1.51	0.981	0.422	
	EX (n=10)	-3.35	4.16			
	BD (n=11)	0.07	7.42			
	EXBD (n=11)	-0.97	1.26			
△TXA_2 (pg/ml)	CON (n=10)	-2.30	2.36	7.227	0.002††	CON>EX** CON>EXBD**
	EX (n=10)	1.52	1.40			
	BD (n=11)	-0.06	1.59			
	EXBD (n=11)	1.86	1.54			

CON=Control, EX=Exercise, BD=Burdock, EXBD=Exercise+Burdock, PGI_2=Prostaglandin I_2, TXA_2=Thromboxane A_2. Values are M±SD. Note: †p<0.05, ††p<0.01; difference value than pre-test.

Table S8. Effects of the 16-week aqua exercise on arterial stiffness in older women with metabolic syndrome.

Variable	Group	Pre		Post		Cohen's d Effect size	Main effect	P-value	Post-hoc
		M	SD	M	SD				
AIx (%)	CON (n=10)	33.14	8.09	42.00*	11.15	.91	Time	0.587	
	EX (n=10)	31.50	7.23	36.83	8.50	.68	Group	0.718	
	BD (n=11)	32.60	9.69	33.80	4.71	.16	Time*Group	0.026† (F=3.829)	
	EXBD (n=11)	42.00	11.54	31.50*	10.09	.97			
AIx@75 (%)	CON (n=10)	29.57	6.92	35.57	9.45	.72	Time	0.673	
	EX (n=10)	27.17	4.79	32.50	8.07	.80	Group	0.366	
	BD (n=11)	31.60	8.62	31.20	2.68	.06	Time*Group	0.06	
	EXBD (n=11)	40.00	9.94	32.33*	7.99				
PWV (sec)	CON (n=10)	9.26	1.36	8.81	1.72	.29	Time	0.721	
	EX (n=10)	8.42	1.03	7.82	0.42	.76	Group	0.065	EX<BD* EX<EXBD*
	BD (n=11)	9.76	0.91	10.08	0.49	.44	Time*Group	0.023† (F=3.974)	
	EXBD (n=11)	8.94	1.38	9.93*	1.12	.79			

CON=Control, EX=Exercise, BD=Burdock, EXBD=Exercise+Burdock, AIx=augmentation index, AIx@75=augmentation index corrected for heart rate, PWV=Pulse Wave Velocity. Values are M±SD. Note: †p<0.05. *p<0.05vspre-trial. Effect size range: .20∣≦small<∣.50∣<medium<∣.80∣≦large.

Table S9. Changes in arterial stiffness after the 16-week aqua exercise in older women with metabolic syndrome

Variable	Group	M	SD	Source		
				F-value	P-value	Post-hoc
△Aix (%)	CON (n=10)	-8.86	13.26	3.829	0.026†	CON<EXBD*
	EX (n=10)	-5.33	9.87			
	BD (n=11)	-1.20	7.05			
	EXBD (n=11)	10.50	10.75			
△AIx@75 (%)	CON (n=10)	-6.00	10.31	2.903	0.060	
	EX (n=10)	-5.33	8.07			
	BD (n=11)	0.40	6.99			
	EXBD (n=11)	7.67	10.58			
△PWV (sec)	CON (n=10)	0.44	1.20	3.974	0.023†	EX<EXBD*
	EX (n=10)	0.60	0.63			
	BD (n=11)	-3.24	0.77			
	EXBD (n=11)	-0.99	0.85			

CON=Control, EX=Exercise, BD=Burdock, EXBD=Exercise+Burdock, AIx=augmentation index, AIx@75=augmentation index corrected for heart rate, PWV=Pulse Wave Velocity. Values are M±SD. †$p<0.05$, ††$p<0.01$; difference value than pre-test.

Table S10. Correlation between delta (Δ) values

Pearson's	ΔSBP	ΔDBP	ΔGlucose	ΔInsulin	ΔHOMAIR	ΔQUICKI	ΔPGI_2	ΔTXA_2	ΔPWV	ΔAIx	ΔAIx@75
ΔSBP	1										
ΔDBP	.657***	1									
ΔGlucose	.035	-.300	1								
ΔInsulin	.056	-.298	.674***	1							
ΔHOMAIR	.128	-.173	.860***	.892***	1						
ΔQUICKI	-.032	.296	-.564**	-.760***	-.608**	1					
ΔPGI_2	-.330	-.283	-.083	.008	-.038	.045	1				
ΔTXA_2	.269	.144	.592**	.201	.427*	-.287	-.371	1			
ΔPWV	-.066	.039	.050	-.136	-.007	.134	-.272	.082	1		
ΔAIx	.070	-.330	.213	.465*	.361	-.411*	.209	.175	-.622***	1	
ΔAIx@75	-.061	-.326	.170	.461*	.359	-.323	.312	.131	-.610**	.964***	1

Delta (Δ): post-pre, SBP: Systolic Blood Pressure, DBP: Diastolic Blood Pressure, HOMA-IR: Homeostatic Model Assessment for Insulin Resistance, QUICKI (Quantitative insulin sensitivity check index), Aix: Augmentation index, Aix@75: Augmentation index corrected for heart rate, PWV: Pulse Wave Velocity. Note: *p<.05, **p<.01, ***p<.001.
